# Relation between Cord Blood Mercury Levels and Early Child Development in a World Trade Center Cohort

**DOI:** 10.1289/ehp.10831

**Published:** 2008-03-31

**Authors:** Sally Ann Lederman, Robert L. Jones, Kathleen L. Caldwell, Virginia Rauh, Stephen E. Sheets, Deliang Tang, Sheila Viswanathan, Mark Becker, Janet L. Stein, Richard Y. Wang, Frederica P. Perera

**Affiliations:** 1 Columbia Center for Children’s Environmental Health, Mailman School of Public Health, Columbia University, New York, New York, USA; 2 Centers for Disease Control and Prevention, Atlanta, Georgia, USA; 3 Center for International Earth Science Information Network, Columbia University, New York, New York, USA; 4 Beth Israel Medical Center, New York, New York, USA

**Keywords:** birth weight, child development, fish consumption, mercury, pregnancy, WTC

## Abstract

**Objective:**

This study was designed to determine whether prenatal mercury exposure, including potential releases from the World Trade Center (WTC) disaster, adversely affects fetal growth and child development.

**Methods:**

We determined maternal and umbilical cord blood total mercury of nonsmoking women who delivered at term in lower Manhattan after 11 September 2001, and measured birth outcomes and child development.

**Results:**

Levels of total mercury in cord and maternal blood were not significantly higher for women who resided or worked within 1 or 2 miles of the WTC in the month after 11 September, compared with women who lived and worked farther away. Average cord mercury levels were more than twice maternal levels, and both were elevated in women who reported eating fish/seafood during pregnancy. Regression analyses showed no significant association between (ln) cord or maternal blood total mercury and birth outcomes. Log cord mercury was inversely associated with the Bayley Scales of Infant Development psychomotor score [Psychomotor Development Index (PDI)] at 36 months (*b* = –4.2, *p* = 0.007) and with Performance (*b* = –3.4, *p* = 0.023), Verbal (*b* = –2.9, *p* = 0.023), and Full IQ scores (*b* = –3.8, *p* = 0.002) on the Wechsler Preschool and Primary Scale of Intelligence, Revised (WPPSI-R), at 48 months, after controlling for fish/seafood consumption and other confounders. Fish/seafood consumption during pregnancy was significantly associated with a 5.6- to 9.9-point increase in 36-month PDI, and 48-month Verbal and Full IQ scores.

**Conclusions:**

Blood mercury was not significantly raised in women living or working close to the WTC site in the weeks after 11 September 2001. Higher cord blood mercury was associated with reductions in developmental scores at 36 and 48 months, after adjusting for the positive effects of fish/seafood consumption during pregnancy.

Mercury can be present in the body as inorganic mercury (mostly Hg^2+^), metallic mercury (elemental, Hg^0^), and organic mercury (mostly methylmercury). Common inorganic sources include air and water pollution, some skin creams, and herbal medicines. Seafood (including fish) consumption is a major contributor of methylmercury to blood total mercury; metallic mercury typically comes from amalgam dental fillings and industrial exposure. Although inhaled metallic mercury is a less common source of blood mercury, in humans about 80% of inhaled metallic vapor is retained by the body [World Health Organization (WHO) Task Group 1991]. It has a half-life in blood of about 60 days (range, 31–100) (Counter and Buchanan 2004). Like methyl-mercury, after crossing the blood–brain barrier, metallic mercury can be oxidized to inorganic mercury, which does not freely return to the circulation, resulting in accumulation in the brain. Much more mercury ends up in the brain of mice and monkeys after inhalation of the metallic form than after injection of the inorganic form (WHO Task Group 1991).

The collapse of the World Trade Center (WTC) towers on 11 September 2001 resulted in the dispersion of many potentially harmful pollutants, including heavy metals (Lioy et al. 2002; McGee et al. 2003; Offenberg et al. 2003). Hundreds of thousands of gallons of airplane fuel, stored diesel fuel, and gasoline in vehicles parked beneath the buildings burned there, releasing some mercury into the air. Metallic mercury was also released with the crushing of tens of thousands of fluorescent light bulbs, major brands of which contain 3.5–15 mg of metallic mercury in a 4-foot bulb (Inform, Inc. 2003). In addition, nearly half of the demolished automobiles would have contained at least one hood or trunk light, or antilock braking system switch bearing metallic mercury (Adsit et al. 2002), each containing about 1,000 mg of metallic mercury [U.S. Environmental Protection Agency (EPA) 2005]. Other possible mercury sources included cathode ray tubes, such as those used for computer screens, and industrial switches and relays, which can contain from 1 to 91,000 g of metallic mercury (U.S. EPA 2002).

Maternal exposure to methylmercury has been associated with decrements in cognitive function in the child (Budtz-Jorgensen et al. 2007; Grandjean et al. 1997, 2005; Jedrychowski et al. 2006; Oken et al. 2005). The developmental effects of maternal inhalation of low levels of metallic mercury vapor during pregnancy have been explored primarily in animals [reviewed by Counter and Buchanan (2004)]; however, in a study of women in Tagum, the Philippines, a fish-eating community using metallic mercury in gold mining/processing, cord blood total mercury was associated with developmental and language deficits at 2 years of age (Ramirez et al. 2003). A few studies have shown a relationship between occupational mercury exposure and various adverse reproductive outcomes using work setting or hair levels to define exposure (Seidler et al. 1999; Sikorski et al. 1986). Less is known about the relation to birth outcomes of lower-level, nonoccupational, metallic mercury exposure. A study, using questionnaires to assess exposure, of pregnant women having mercury dental fillings replaced during pregnancy showed no reductions in newborn birth weight (Hujoel et al. 2005).

To evaluate the relation of blood total mercury to birth outcomes and child development, we used data collected in a study of the effects of the WTC attack of 11 September 2001 on women delivering several months after the event. We measured total mercury in maternal and cord blood, a measurement that reflects methylmercury from fish consumption, as well as inorganic and metallic mercury. We explored the relation of blood total mercury to proximity to the WTC and hypothesized that higher blood total mercury levels, whatever their source, would be associated with differences in birth outcomes and adversely affect child development, as measured by the Bayley Scales of Infant Development (2nd ed., BSID-II) (Bayley 1993) at 12, 24, and 36 months, and the Wechsler Preschool and Primary Scale of Intelligence, Revised (WPPSI-R) (Wechsler 1989), at 48 months.

Although consumption of fish has been shown to be associated with higher maternal and/or cord mercury concentrations (Daniels et al. 2004; Jedrychowski et al. 2006), significantly higher infant scores on the MacArthur Communicative Development Inventory at 15 months and on the Denver Developmental Screening Test at 18 months have been associated with increasing fish intake during pregnancy (Daniels et al. 2004). Therefore, we also evaluated whether maternal fish/seafood consumption would have a protective effect on the child’s cognitive development and whether, by including it in the analyses, we would more clearly detect detrimental effects of prenatal mercury exposure.

## Subjects and Methods

### Sample selection

Participants were recruited at Beth Israel and St. Vincent’s Hospitals (and St. Vincent’s–affiliated Elizabeth Seton Childbearing Center), both approximately 2 miles from the WTC site, and at New York University Downtown Hospital, within a half-mile of the site. All three hospitals draw women from Manhattan, the outlying New York City boroughs, and parts of New Jersey and Westchester, New York. Enrollment was implemented at each hospital as soon after 11 September as its institutional review board approval was obtained. Women were approached for enrollment when they presented for labor and delivery between 12 December 2001 and 26 June 2002. Women were briefly screened for eligibility [nonsmokers, between 18 and 39 years of age, bearing a single infant, and meeting geographic and health criteria detailed by Lederman et al. (2004)], recruited, enrolled, gave informed consented before delivery, and were interviewed after delivery, all in their preferred or native language (English, Spanish, or Chinese). Of 738 women initially approached, 240 women were ineligible and 129 refused to participate. Of 369 eligible women who gave signed consent for participation, 329 contributed cord or maternal blood or both, a complete postpartum interview, and medical record data, all of which were required for final enrollment.

### Biological sample analyses

Hospital staff collected cord blood at delivery and maternal blood generally within 24 hr after delivery. Samples were transported to our laboratory at Columbia Presbyterian Medical Center in New York City for processing. Aliquots were stored at –70°C and later shipped to the environmental health laboratory at the Centers for Disease Control and Prevention (CDC) in Atlanta, Georgia. Analyses for whole-blood total mercury (inorganic, metallic, and organic mercury) were conducted at the CDC using inductively coupled plasma mass spectrometry. Blood samples were diluted with ≥ 18 MΩ water and with diluent containing 1% vol/vol tetramethylammonium hydroxide, 0.5% disodium ethylenediamine tetraacetate, 10% ethyl alcohol, and 0.05% Triton X-100. Gold was added to reduce intrinsic mercury memory effects, and bismuth for internal standardization of mercury (Barany et al. 1997; Date and Gray 1989; Nixon et al. 1999). The samples were prepared with a 1:1:48 ratio of sample:water:diluent (CDC 2003). Of 286 cord and 212 maternal samples collected, one cord and six maternal blood samples were less than the limit of detection (LOD); they were assigned a value of LOD/2.

### Data collection

Pregnancy and delivery information were collected from the medical records of the mother and newborn after delivery. During the postpartum interview, detailed residential and employment addresses, for the locations where the woman actually lived or worked, were obtained for each of the 4 weeks after 11 September. These were geocoded at the Center for International Earth Science Information Network of Columbia University’s Earth Institute, using geographic information system software from Environmental Systems Research Institute (Redlands, CA), including ArcGIS 8.3, the Street Map 2003 extension, and the New York City Department of Environmental Protection Map. Geocoded addresses were used to compute the linear distance from the WTC site, with an estimated horizontal accuracy of ± 25 feet. Information was obtained on maternal demographics, material hardship (inability to pay for essentials; Mayer and Jencks 1989), education, household income, exposure to environmental tobacco smoke (ETS), employment history, housing characteristics, and types of fish or seafood consumed during pregnancy. Fish/seafood eaters were classified according to the number of different types of fish/seafood reported consumed, up to a maximum of three types. Frequency of fish/seafood meals, levels of mercury in different fish/seafood, and quantity consumed per meal were not considered in these classifications.

Women were reinterviewed at about 6-month intervals after delivery through child’s age of 4 years. These interviews included questions concerning the duration and exclusiveness of breast-feeding. At the annual time points, the children were invited to our offices for measurement of weight, height, and head circumference and for assessment of growth and development by specially trained interviewers. The Mental Development Index (MDI) and the Psychomotor Development Index (PDI) scores from the BSID-II administered to the children at about 12, 24, and 36 months of age, and the Performance, Verbal, and Full IQ scores from the WPPSI-R administered at about 48 months were used as the developmental outcomes. We used the WPPSI-R (Wechsler 1989), rather than the third edition (Wechsler 1991), because of its availability in Chinese. If necessary, assessments were performed at the child’s home, when that was feasible. Not all mothers agreed to have their child followed after birth (many of the Chinese children were to be raised in China), and not all children were available for all developmental assessments. Only children delivered at term (> 258 days) and conceived by 11 September 2001 were included in the developmental analyses. For subjects meeting these criteria, MDI scores were completed at 12, 24, and 36 months of age for 151, 151, and 140 children, respectively; PDI scores, for 151, 149, and 136 children, respectively; and the WPPSI-R at 48 months for 130 children.

The BSID-II, a widely used, norm-referenced developmental test, can be used to diagnose developmental delay and has been shown to be sensitive to the effects of low-level intrauterine exposure to mercury (Jedrychowski et al. 2006) and lead (Bellinger et al. 1987). Each scale provides a developmental quotient (raw score/chronologic age), generating a continuous MDI or PDI score, both with mean ± SD of 100 ± 15. When administered at 36 months of age, the BSID-II demonstrates moderate predictive power for subsequent intelligence and school performance, and it is clinically useful for identifying children performing in the subnormal range (Bayley 1993; Burchinal et al. 2000; Sternberg et al. 2001). Children are classified as normal (≥ 85), moderately delayed (< 85 to > 70), or severely delayed (≤ 70) on the BSID-II. On the WPPSI-R (standardized mean, 100 ± 15), a score of 89–80 is low average, 79–70 is borderline, and ≤ 69, intellectually deficient. During the child’s assessment visits, maternal intelligence was measured by the Test of Non-verbal Intelligence, 2nd edition (TONI; Brown et al. 1990), a 15-min, language-free measure of general intelligence, relatively stable and free of cultural bias. The mean of two values was available for 53% of the women who were tested at two visits; eight mothers did not attend any visit and were not tested.

### Statistical analyses

We examined descriptive statistics and bivariate associations for all variables for distributional normality; if needed, variables were natural log–transformed. In analyses examining relationships between proximity to the WTC site and mean blood total mercury, we classified subjects into three “distance” groups: those who at some time in the 4 weeks after 11 September resided within 2 miles of the WTC site (residential group), those who worked within 2 miles of the WTC at some time during this period but did not reside there (employed group), and those who neither worked nor resided within this radius (reference group). For some analyses, a 1-mile radius was used to create three analogous groups. Women in the residential groups included some women who were employed. Location of their employment was not considered in their classification.

We tested differences in mean maternal and cord blood total mercury levels of the three distance groups by Kruskal–Wallis and Mann–Whitney tests. The relation of blood mercury to proximity to the WTC site was evaluated for each of the 4 weeks after 11 September using Spearman’s correlation with a continuous measure of distance from the WTC site, excluding subjects living > 20 miles from the WTC site. Mercury levels were not normally distributed, and their variances differed, so we used log-transformed blood mercury levels in an ordinary least squares (OLS) regression to identify a linear trend across the residential, employed, and reference groups.

Cord blood mercury is considered a better measure of fetal exposure than maternal blood (Grandjean et al. 2005) or hair mercury (Budtz-Jorgensen et al. 2004); we also had more cord than maternal blood samples, and more of them had total mercury levels greater than the LOD. Therefore, we used cord mercury for all outcome analyses and used maternal blood total mercury only for selected analyses. We used multiple regression to assess associations of blood mercury with birth weight, length, and head circumference and with subsequent development as measured by BSID-II and WPPSI-R scores, adjusting for confounding factors possibly influencing child development. We used logistic regression to estimate associations between log cord blood mercury and the likelihood of scoring in the delayed or severely delayed ranges on these tests. Statistical analyses used SPSS software (version 14; SPSS Inc., Chicago, IL) and two-tailed tests; *p* ≤ 0.05 indicated statistical significance. We express data as means ± SD.

## Results

### Participants

The 329 participants resided and worked throughout the New York City area [see map of locations in Lederman et al. (2004)]. We obtained cord blood from 94.7% of newborns of Asian women and 82.9% of those of non-Asian women, and maternal blood from 31.8% of Asians and 81.5% of non-Asians. Blood donation is a cultural taboo in China (Gill et al. 2002), and most of the Chinese participants provided only a cord blood sample. To check for possible bias introduced by missing data, we compared women with and without mercury measurements in cord blood. No significant differences were found for maternal age, household income per family member, education (less than high school, high school diploma, more than high school), race/ethnicity (Asian, black, white, other/not reported), and ETS exposure at home during pregnancy, using the Mann–Whitney or chi-square analysis. In contrast, the smaller group of women with a maternal blood sample (and mercury measurement) differed from those without that measurement on race/ethnicity, income, education, and maternal age, because of the lower proportion of samples from Asians, as indicated by the lack of significant differences in women with and without maternal mercury measurements within the Asian and non-Asian subgroups considered separately.

### Blood total mercury

In 163 paired samples, mean mercury level was significantly higher in cord than in maternal blood (5.05 ± 6.64 μg/L vs. 2.29 ± 2.33 μg/L, respectively; *p* < 0.01, paired Wilcoxon signed rank test). Levels in paired samples were also significantly correlated (Spearman’s rho = 0.83, *p* < 0.01). Paired blood mercury levels were significantly higher in women who ate fish/seafood during pregnancy (cord = 5.10 ± 12.44 μg/L; maternal = 2.59 ± 2.50 μg/L, *n* = 114) than in women who did not (cord = 2.97 ± 2.89 μg/L; maternal = 1.67 ± 1.78 μg/L; *n* = 54; *p* < 0.001 for both comparisons by Mann–Whitney, including values below the LOD). Cord mercury was correlated with consumption of fish/seafood, whether coded yes/no or as number of different types eaten during pregnancy [correlation coefficients = 0.28 (*p* = 0.000) and 0.164 (*p* = 0.006), respectively].

Table 1 shows data for blood mercury concentration in all (unpaired) cord and maternal samples. China-born Asian women had much higher cord blood total mercury than did non-Asians or Asians born elsewhere (only one was U.S. born). This remained true when the women were classified by number of types of fish/seafood eaten (Table 2). Almost one-third of the women (31.6%) reported no fish/seafood consumption during pregnancy.

Table 1 shows the ratio of cord mercury to maternal mercury (2.18 ± 1.72), excluding cases with either value less than the LOD. The ratio was not significantly different by *t*-test for China-born Asians (2.27 ± 0.79) versus non-China-born Asians (2.70 ± 2.71) but was significantly higher for fish/seafood eaters (2.08 ± 2.76, *n* = 112) compared with non-seafood eaters (1.97 ± 1.50; *n* = 51; *p* = 0.020 by Mann-Whitney test). The ratio was not significantly different between women residing within 2 miles of the WTC site (1.65 ± 3.75) compared with women working within 2 miles of the site (2.16 ± 1.18) or in the reference group (2.20 ± 1.95)

### Relationship between blood mercury and proximity to the WTC site

In the first through fourth weeks after 11 September, 81, 78, 80, and 81 women, respectively, lived within 2 miles of the WTC, and 15, 16, 17, and 18, respectively, within 1 mile. Women whose homes were in the restricted-access area were generally away from their homes throughout the whole 4 weeks and thus would not be included in our residential group. The corresponding numbers for those 4 weeks for women only working within 2 miles were 36, 62, 72, and 73, and for those working within 1 mile, 14, 37, 44, and 46. These numbers show that most of our participants who resided near the WTC were there in the first week or two after 11 September; they also spent more time there each day (16.7 hr/day), compared with those who were only employed near the WTC [7.7 hr/day; for details, see Lederman et al. (2004)]. Air pollution may also have been greater during the evening compared with during working hours (Lederman et al. 2004). Thus, women in the residential group were closer and had more hours of potentially more intense exposure, compared with women in the employed or reference groups.

We compared unadjusted mean cord and maternal blood mercury in the three groups defined by distance of the women’s residences and work sites from the WTC during the first 4 weeks after 11 September (Table 3). Neither cord nor maternal blood total mercury was significantly higher in the women residing or working within 1 or 2 miles of the WTC compared with their respective reference groups; cord mercury in the employed group was significantly lower than in the residential group. Neither cord nor maternal blood mercury was significantly correlated with a continuous measure of linear distance of the mother’s residence from the WTC site in any week (excluding women residing > 20 miles away) or when the analysis was limited to those residing within 1 or 2 miles of the WTC site (data not shown). There was no significant trend in (log) cord or maternal blood mercury in the three 1-mile or 2-mile groups by OLS regression.

### Blood mercury, birth measures, and developmental outcomes

Table 4 shows participant characteristics and developmental scores for women whose children were included in the developmental assessments. Mean mercury levels are shown for the categorical variables. Mercury levels were significantly different for cord blood and maternal blood by Medicaid status, fish/seafood consumption, and Asians versus others. Cord levels also differed for blacks versus others; maternal levels differed by material hardship.

There were no significant associations between (log) cord or maternal blood mercury and birth weight, length, head circumference, or gestational duration in linear regression models adjusted for baby’s sex and gestational age (not used for gestational duration), and maternal race, age, Medicaid receipt, prenatal ETS exposure, fish/seafood during pregnancy, parity, trimester of pregnancy on 11 September, prepregnancy weight, height, or pregnancy complications, (and cesarean section for head circumference analyses only).

With multivariable linear regression (Table 5), lncord mercury was not a significant determinant of MDI or PDI at 12 or 24 months, but the inverse relationship between mercury level and test scores increased with age and was significant for 36-month PDI and 48-month Performance, Verbal, and Full IQ. The approximate effect of a doubling of cord mercury on the measured outcome is estimated by multiplying the *b*-value for lncord mercury by 0.69 (ln 2 = 0.69). Thus, a doubling of cord mercury is associated with a decrease of 2.5 points for Full IQ (–3.6 × 0.69); predicted full IQ would be 98.9 at the mean cord mercury level (7.74 μg/L, with those not pregnant on 11 September excluded) and 114 for those at 0.1 μg/L.

We created more parsimonious models for the 36- and 48-month outcomes, including only covariates with *p* < 0.1 (Table 5). In these reduced models, cord mercury remained significantly negatively associated with the 36-month PDI and the performance, verbal, and full IQ at 48 months. Fish/seafood consumption was also associated with significantly increased scores on these tests except for performance IQ. The model generally explained more of the variance as the child aged, explaining 38% in the WPPSI-R Full IQ at 48 months. Linear regressions using the smaller number of maternal blood total mercury samples showed similar, but smaller, negative effects of mercury and positive effects of seafood consumption on child development; none of these was statistically significant (data not shown).

To further explore the relation of fish/seafood consumption and cord blood mercury to development, we examined regressions (not shown) that excluded one or the other of these variables from the reduced models. Although both variables were significantly related to developmental scores at 36 and 48 months when both were included in the model (Table 5), neither was significant with the other eliminated from the model for 36-month PDI or for 48-month Performance or Verbal IQ. For Full IQ, cord blood mercury remained significant (*b* = − 2.57, *p* = 0.025) without fish/seafood consumption in the model, but fish/seafood consumption was not significant without mercury in the model.

Psychosocial stress, possibly due to the occurrence of the 11 September tragedy during pregnancy, could potentially contribute to the poorer developmental outcomes we observed. Therefore, we examined the effect of the two psychosocial factors that we measured (material hardship and demoralization, considered together and separately) in regression analyses of the child’s development at 48 months. We found no independent effect of these psychosocial factors on Performance, Verbal, or Full IQ (all *p*-values > 0.4) in any of these analyses, whereas the relations of fish consumption and cord mercury levels to these outcomes remained virtually unchanged.

We examined the relation of cord blood mercury to developmental delay in logistic regression analyses, controlling for the same variables as used in the MDI and PDI analyses (excluding child’s age at testing for these across-time analyses). Cord blood mercury was not significantly related to the odds of developmental delay at any age when children were classified as delayed based on an MDI or PDI score ≤ 85 or on either a borderline score (≤ 79) or low average score (≤ 89) on the WPPSI, cutoffs suggested in the documentation for these tests. However, no child with a cord mercury level greater than about 13 μg/L had a full IQ score greater than 100.

## Discussion

Estimates of the ratio of cord to maternal blood mercury are used to model fetal exposure based on maternal blood levels and to derive the maternal reference dose. There is a persisting need for data on the variability of this ratio (Rice et al. 2003). Our mean ratio (2.18 ± 1.72) is consistent with mean values for 10 studies summarized in a recent review (Stern and Smith 2003), which ranged from 1.09 to 2.32. Higher cord blood mercury may be due to greater binding of mercury by fetal hemoglobin, higher fetal hemoglobin concentrations, and continued fetal exposure from repeated swallowing of urine in amniotic fluid and from intestinal resorption of mercury from fetal feces. Maternal mercury is lost in urine and feces.

We were unable to demonstrate significant increases in mercury levels because of residential or work-site proximity to the WTC site in the weeks after 11 September. Although mercury may have been released during the WTC tragedy, effects of that exposure may have been obscured by the short half-life of metallic mercury in blood, the collection of blood months after the event, our lack of inorganic mercury measures, the high and variable contribution of methylmercury from seafood, and possibly mercury from earlier environmental exposures, especially among our China-born women, whose levels were very high. Nevertheless, the relation of blood total mercury to later child development does not depend on the source of blood mercury.

We found no significant relationship of total mercury in cord or maternal blood to newborn size or gestational age at birth. In contrast, the relation to child development was clear and increased between the ages of 1 and 3 years. The (log) mercury level in cord blood was associated with a significant, –4.2 point decrement in the 36-month PDI of the BSID-II and with decrements of –3.4, –2.9, and –3.8 points in Performance, Verbal, and Full IQ, respectively, on the WPPSI-R at 48 months, in regression models that controlled for selected determinants/confounders. Several previous studies reporting adverse effects of mercury on cognitive development have studied populations with higher levels of mercury than in our cohort, largely from methylmercury (e.g., Grandjean et al. 1997: cord total mercury geometric mean = 22.9 μg/L in Faroe Island children; present study = 4.4 μg/L).

In the present study, maternal blood mercury was similar to values from the 1999–2002 National Health and Nutrition Examination Survey (Jones et al. 2004), where the geometric mean for women of child-bearing age was 0.92 μg/L (present study, 0.91 μg/L), with 5.66% (present study, 5.95%) having levels ≥ 5.8 μg/L. Our median cord blood total mercury level was 4.3 μg/L (32.1% ≥ 5.8 μg/L), compared with 0.85 μg/L in a recent Polish study (Jedrychowski et al. 2006). In a Swedish study (Bjornberg et al. 2003), median cord blood methylmercury was 1.3 μg/L (range, 0.1–5.7 μg/L), with inorganic mercury about one-tenth as high (0.15 μg/L; range, 0.03–0.53 μg/L). Despite the difference in levels, all three studies reported a positive association between blood mercury levels and fish consumption, whether measured as number of types of fish/seafood consumed during pregnancy (present study), maternal fish/seafood consumption in the year preceding pregnancy (Bjornberg et al. 2003), or estimated weekly fish consumption during pregnancy (Jedrychowski et al. 2006).

We observed higher mercury levels in China-born Asians than in the other ethnic groups. Higher hair mercury levels, even after controlling for fish/seafood consumption, have been reported previously in Asians compared with non-Asians recruited from across the United States (Patch et al. 2005). Differences in frequency of fish/seafood meals or in the amount consumed at each such meal could be partly responsible for differences in blood mercury levels in our ethnic groups, because we used only the number of different types of fish/seafood eaten during pregnancy to classify women; however, even among those not eating seafood, China-born Asians had higher mercury levels than did other groups.

The cord:maternal mercury ratio was significantly higher for fish eaters than for non-fish eaters, whereas it was not higher for China-born than for non-China-born Asian women. These results support the belief that the high mercury levels of China-born Asians were at least partly related to factors other than seafood consumption, such as from pollution in China from mercury-containing coal used for energy production (Pottinger et al. 2004) or from use of herbal drugs that contain mercury (California Poison Action Line 2002).

It is noteworthy that, in reduced models with mercury level controlled, maternal fish/seafood consumption during pregnancy was associated with an 8.7-point increase in PDI score at 36 months and a 5.6 point increase in Verbal and Full IQ scores at 48 months, suggesting the value of consuming fish/seafood during pregnancy. Recent work reanalyzing data collected in the 1980s in the Faroe Islands (Budtz-Jorgensen et al. 2007) has demonstrated the importance of considering the positive effects of maternal fish intake on child development at 7 and 14 years of age, when estimating the adverse effects of high methylmercury exposure. In that study, fish intake had the greatest positive effect on motor function outcomes, consistent with our observation of a positive relation of fish/seafood consumption to the child’s PDI score at age 3 years of age. A beneficial effect of fish consumption on BSID-II cognitive and psychomotor scores at 1 year of age, with a measurable negative effect of (low) blood mercury levels, has also been reported (Jedrychowski et al. 2006), as has a positive relation of fish consumption to a Visual Recognition Memory score at 6 months of age, with a negative relation to (low levels of) hair mercury (Oken et al. 2005).

These studies, combined with ours, show positive developmental effects of maternal fish/seafood consumption and negative effects of blood mercury at six different ages (0.5, 1, 3, 4, 7, and 14 years) over a very wide range of mercury levels. Apparently, maternal consumption of fish/seafood can raise fetal mercury levels yet enhance cognitive development in the child, probably because of other constituents, particularly omega-3 fatty acids (Innis 2004). Thus, inclusion of a measure of maternal fish/seafood intake appears to be important for demonstrating the true magnitude of the detrimental effect of low levels of mercury on development.

## Conclusion

We were unable to demonstrate significantly higher blood total mercury levels in women residing or working close to the WTC site in the 4 weeks after 11 September 2001. The range of maternal blood mercury we observed was typical of values seen in national studies, with cord levels more than twice maternal levels. Of concern are the reductions in psychomotor development (PDI) at 36 months and in Performance, Verbal, and Full IQ at 48 months that were associated with increased cord mercury in these women. The strong positive association observed between child development and maternal fish/seafood consumption during pregnancy, combined with the large portion of our women reporting no fish/seafood consumption (> 30%), and the consistency of these findings with other recent studies over a broad range of child ages and mercury levels, indicates that pregnant women should be encouraged to consume fish/seafood while guided to reduce intake of varieties high in mercury.

## Figures and Tables

**Table 1:** Blood total mercury (μg/L) and ratio of cord to maternal blood mercury.

	All subjects		China-born Asian		Asian, not China born		Non-Asian		All subjects	
	Cord Hg	Maternal Hg	Cord Hg	Maternal Hg	Cord Hg	Maternal Hg	Cord Hg	Maternal Hg	Cord:maternal Hg ratio^a^
Mean	7.82	2.32	17	6.01	5.97	2.69	3.73	1.87	2.18
SD	9.71	2.3	13	4.65	4.88	2.45	3.33	1.33	1.72
95% CI	6.67–8.96	2.01–2.63	14.2–19.8	3.83–8.18	3.62–8.32	1.38–3.99	3.24–4.22	1.67–2.06	1.92–2.45
Median	4.3	1.7	15.8	4.0	4.6	1.95	2.9	1.6	1.87
Geometric mean	4.44	1.6	12.6	4.54	4.18	1.74	2.74	1.41	1.94
95% CI	3.91–5.04	1.41–1.81	10.56–15.12	3.17–6.49	2.65–6.60	0.996–3.04	2.43–3.10	1.20–1.59	1.81–2.07
Minimum	0.10^b^	0.07^b^	1.0	1.6	0.4	0.3	0.10^b^	0.07^b^	0.4
Maximum	63	16.4	63	16.4	18.2	9.3	30	7.5	18.75
No.	280	212	83	20	19	16	178	176	163

a Only paired samples for cord:maternal ratio; excludes samples with mercury less than the LOD.

b These values represent one-half the LOD.

**Table 2:** Maternal and cord blood total mercury by number of types of fish/seafood reported eaten during pregnancy.

		Maternal Hg			Cord Hg		
Race/ethnicity	No. of fish/seafood types reported	μg/L	No. of measures	SD	μg/L	No. of measures	SD
Asian	0	2.87	12	3.12	14.00	28	15.15
	1	6.71	10	3.68	15.90	39	9.82
	2	5.44	8	6.10	16.41	24	15.03
	≥ 3	3.02	6	1.19	10.81	11	9.15
	Total	4.53^a^	36	4.13	14.95^a^	102	12.66
White	0	1.36	33	1.07	2.18	38	1.63
	1	1.59	20	1.04	3.68	17	3.53
	2	2.06	21	1.35	4.06	18	2.03
	≥ 3	2.55	35	1.56	4.84	35	3.28
	Total	1.92	109	1.37	3.59	108	2.85
Black	0	1.48	12	1.04	2.47	12	2.13
	1	1.41	7	0.73	2.47	6	1.41
	2	1.75	13	0.98	4.10	16	2.07
	≥ 3	2.32	9	2.11	4.38	11	2.75
	Total	1.74	41	1.29	3.52	45	2.30
Other/not reported	0	1.24	10	0.55	1.82	6	1.09
	1	2.15	9	1.75	6.52	10	8.88
	2		0		4.85	2	2.76
	≥ 3	2.34	7	1.04	4.61	7	2.16
	Total	1.85	26	1.26	4.72	25	5.90
All non-Asians combined	0	1.36	55	0.98	2.20	56	1.68
	1	1.70	36	1.21	4.32	33	5.57
	2	1.94	34	1.21	4.11	36	2.01
	≥ 3	2.48	51	1.59	4.71	53	3.00
	Total	1.87^a^	176	1.33	3.73^a^	178	3.33

a Group means for maternal and cord blood mercury were significantly different for Asians compared with all non-Asians (both *p* < 0.01, Mann–Whitney).

**Table 3:** Cord and maternal blood mercury levels [mean ± SD (no.)] by proximity of residence or employment location to the WTC site in the 4 weeks after 11 September 2001.

Group	Cord total Hg (*n*)	Maternal total Hg (*n*)
Within 2 miles of the WTC in the 4 weeks after 11 September		
Resided	9.46 ± 11.28^a^,^b^ (73)	2.48 ± 2.42 (54)
Employed	4.67 ± 4.40^a^,^b^ (46)	2.23 ± 2.31 (45)
Reference	7.97 ± 9.88^a^ (161)	2.27 ± 2.26 (113)
Within 1 mile of the WTC in the 4 weeks after 11 September		
Resided	10.6 ± 11.4 (18)	3.4 ± 2.8 (12)
Employed	5.7 ± 5.5 (35)	2.6 ± 2.8 (34)
Reference	7.9 ± 10.0 (227)	2.2 ± 2.1 (166)

No significant trend in log-transformed cord or maternal mercury in the three 1- or 2-mile groups (OLS regression); no significant difference in maternal mercury in the three 1- and 2-mile groups, Kruskal–Wallis Test.

a *p* = 0.047 for difference in unadjusted log cord mercury in the three 2-mile groups, Kruskal–Wallis test.

b *p* = 0.013 for difference in unadjusted log cord mercury between residential and employed group, Mann–Whitney test.

**Table 4:** Participant characteristics.^a^

Variable	No.	Mean ± SD	Category	Cord blood Hg (μg/L)	Maternal blood Hg (μg/L)
Categorical variable					
Male child	151	49.0 ± 50.2^b^	Female	5.80 ± 6.74	2.14 ± 1.90
			Male	5.83 ± 6.81	2.15 ± 1.87
Asian	151	27.2 ± 44.6^b^	Asian	10.59 ± 9.89#	3.54 ± 3.16#
			Non-Asian	4.03 ± 3.87	1.89 ± 1.44
Black/African American	151	18.5 ± 39.0^b^	Black	3.31 ± 2.25**	1.73 ± 1.51
			Non-black	6.38 ± 7.29	2.25 ± 1.96
Married or cohabiting ≥ 7 years	151	81.5 ± 39.0^b^	Married	5.54 ± 5.93	2.23 ± 1.98
			Nonmarried	6.80 ± 9.49	1.78 ± 1.38
ETS in home during pregnancy	151	19.9 ± 40.0^b^	Yes	5.99 ± 7.00	1.58 ± 0.80
			No	5.77 ± 6.71	2.26 ± 2.02
Ate fish/seafood during pregnancy	151	71.5 ± 45.3^b^	Yes	6.70 ± 6.99#	2.41 ± 2.06**
			No	3.60 ± 5.56	1.54 ± 1.19
Parity (≥ 1)	151	35.1± 47.9^b^	≥ 1	6.28 ± 8.36	2.14 ± 2.02
			0	5.56 ± 5.72	2.14 ± 1.81
Medicaid (yes/no)	47	31.1± 46.4^b^	Yes	8.94 ± 10.03**	2.79 ± 2.86*
			No	4.40 ± 3.86	1.94 ± 1.41
Material hardship (yes/no)	151	6.6 ± 25.0^b^	Yes	6.04 ± 3.56	3.34 ± 2.30**
			No	5.80 ± 6.93	2.03 ± 1.81
Noncategorical variable					
Per capita household income ($10k units)	141	2.68 ± 1.75			
Education, years	151	14.8 ± 3.5			
Maternal IQ, TONI	143	96.8 ± 14.6			
Child’s proportion breast-fed, first year^c^	147	0.287 ± 0.296			
Age (days) at testing					
At 12-month testing	151	393.8 ± 42.0			
At 24-month testing	133	739.6 ± 28.2			
At 36-month testing	116	1,106 ± 47.4			
At 48-month testing	107	1,486 ± 39.2			
MDI score					
12 months	151	95.7 ± 8.04			
24 months	129	96.1 ± 12.6			
36 months	115	96.6 ± 10.5			
PDI score					
12 months	151	99.9 ± 12.7			
24 months	128	97.3 ± 10.9			
36 months	112	98.4 ± 13.1			
48-month WPPSI-R IQ					
Performance	107	100.7 ± 13.9			
Verbal	107	95.7 ± 13.2			
Full	107	97.6 ± 12.7			

a Includes participants delivered after 258 days of gestation, pregnant on 11 September, with completed 12-month child assessment.

b Mean percent ± SD.

c Proportion breast-fed for first year was computed from 6-, 12-, and 18-month interview reports as (days exclusive breast-feeding, first year, + 50% of days of mixed feeding, first year) divided by 365, where exclusive breast-feeding = no other milks/formula reported used during a breast-feeding day, and mixed feeding = other milks/formula and breast milk used on the same day.

* *p* < 0.05,

** *p* < 0.02,

# *p* < 0.005, significant differences for contrasts within variable, using Mann–Whitney test.

**Table 5:** Full and reduced linear regression models for developmental outcomes at ages 36 and 48 months.

		Full regression model					Reduced regression model				
Measure	Statistic	Ate fish/seafood during pregnancy	Lncord mercury	Adjusted *R*^2^	Model *p*-value	No.	Ate fish/seafood during pregnancy	Ln-cord mercury	Adjusted *R*^2^	Model *p*-value	No.
MDI 12	*b*	2.49	−0.53	−0.010	0.558	132	2.68	−0.81	−0.008	0.548	133
	*p*	0.192	0.600				0.148	0.407			
PDI 12	*b*	2.36	−1.39	0.008	0.390	132	2.15	−1.75	0.011	0.314	133
	*p*	0.408	0.358				0.436	0.231			
MDI 24	*b*	2.99	−2.38	0.234	0.000	131	2.44	−2.76	0.214	0.000	132
	*p*	0.231	0.072				0.325	0.035			
PDI 24	*b*	4.58	−2.20	0.069	0.069	130	3.77	−2.56	0.019	0.244	131
	*p*	0.070	0.101				0.138	0.055			
MDI 36	*b*	4.13	−1.43	0.092	0.046	114	4.21	−1.32	0.112	0.008	114
	*p*	0.092	0.236				0.080	0.26			
PDI 36	*b*	9.22	−4.07	0.114	0.025	111	8.73	−4.16	0.150	0.002	111
	*p*	0.005	0.010				0.006	0.007			
Performance IQ	*b*	4.07	−3.20	0.180	0.003	107	4.26	−3.45	0.200	0.000	107
	*p*	0.161	0.039				0.137	0.023			
Verbal IQ	*b*	5.60	−2.87	0.351	0.000	107	5.60	−2.92	0.370	0.000	107
	*p*	0.025	0.030				0.022	0.023			
Full IQ	*b*	5.54	−3.616	0.372	0.000	107	5.64	−3.76	0.385	0.000	107
	*p*	0.019	0.004				0.015	0.002			

Reduced models controlled for race (Asian, black, other), maternal IQ (TONI), per capita family income, and child’s sex and gestational age at birth. Full models controlled for these variables plus maternal age, prenatal ETS exposure, marital status, education, material hardship, proportion breast-feeding (see Table 4, note *c*), and exact age in days at testing.
